# Effects of Exergaming on Preschoolers’ Executive Functions and Perceived Competence: A Pilot Randomized Trial

**DOI:** 10.3390/jcm8040469

**Published:** 2019-04-06

**Authors:** Shanying Xiong, Peng Zhang, Zan Gao

**Affiliations:** 1 Department of Physical Education, Shenzhen Polytechnic University, Shenzhen 518055, China; 2 College of Health Sciences, East Stroudsburg University of Pennsylvania, East Stroudsburg, PA 18301, USA; pzhang@po-box.esu.edu; 3 School of Kinesiology, University of Minnesota-Twin Cities, Minneapolis, MN 55455, USA; gaoz@umn.edu

**Keywords:** active video games, cognitive functions, perceived physical competence, perceived social acceptance, preschool children

## Abstract

**Purpose:** This study aimed to evaluate the effects of a child-centered exergaming program and a traditional teacher-led physical activity (PA) program on preschoolers’ executive functions and perceived competence. **Methods:** Sixty children aged 4–5 years from an urban childcare center in China completed an 8-week exergaming/traditional PA intervention. After baseline measurements of executive functions and perceived competence (i.e., perceived physical competence and social acceptance), children were randomly assigned to either an exergaming group or traditional PA group (30 children per group). Exergaming and traditional PA programs were offered 20 min/session by trained instructors for 8 weeks. Post-intervention measures were identical to baseline measures. **Results:** In general, children’s executive functions, perceived physical competence, and perceived social acceptance were enhanced over time. Analysis of variance revealed significant time by group interaction effects for executive functions, *F*(1, 58) = 12.01, *p* = 0.01, and perceived social acceptance, *F*(1, 58) = 6.04, *p* = 0.02, indicating that the exergaming intervention group displayed significantly greater increases in executive functions and perceived social acceptance in comparison with traditional PA children. In addition, children’s executive functions and perceived physical and social competence significantly improved from baseline to post-intervention. However, there was no significant difference in the increase of children’s perceived physical competence across groups over time. **Conclusion:** The results suggested exergaming to be beneficial in enhancing young children’s executive functions and perceived social acceptance compared to the traditional PA program. More diverse samples with a longer intervention duration in preschool children in urban areas are warranted.

## 1. Introduction

Low levels of cardiorespiratory fitness in children may increase the risk of multiple chronic diseases, such as hypertension, hypercholesterolemia, and diabetes [1,2]. Regular participation in physical activity (PA) has been observed to improve children’s health and fitness [3]. Due to an increasing prevalence of childhood obesity among preschool-aged children globally, the preschool years (i.e., 4–5 years) have been identified as a crucial time to promote PA, enhance cardiorespiratory fitness, and optimize overall wellness and development [4]. Yet, research indicates that, on average, preschoolers accumulate only 43 min of moderate-to-vigorous PA/day—well below the recommended 180 min/day of PA participation in this population [5]. Accordingly, early childhood PA promotion strategies are called for to help preschoolers establish physically active lifestyles and foster optimal development [6]. Few studies, however, have investigated the effects of innovative PA interventions on urban preschoolers’ PA and optimal development (e.g., physical growth, cognitive development), especially in developing countries [7]. That is, there is an urgent need to develop, implement, and evaluate developmentally appropriate and innovative PA interventions targeting urban preschool children to promote health and cognitive development in this population. As such, innovative, technology-based PA programs that are fun, motivating, and pervasive for children [8,9] need to be developed and evaluated among preschool-aged children. 

One such PA modality that may achieve this objective may be exergaming (i.e., video games that are also a form of exercise) [10]. Specifically, exergames integrate exercise with the fun component of the gaming entertainment which attracts both youth and adults. That is, exergaming capitalizes on a player’s interest in sedentary-style video games but requires s/he to engage in light-to-moderate level, even a vigorous level, of PA, depending on the type and difficulty level of the exergames [11]. In the past decade, exergaming has been increasingly used in communities and schools as an innovative and fun strategy to promote healthy and active lifestyles [12]. For example, child-centered exergaming has been observed to be effective in promoting elementary school children’s PA levels and maximum oxygen consumption [13,14,15], cardiovascular fitness, motor skills [16,17], and psychosocial beliefs [18]. However, the majority of previous studies have only targeted older children and adolescents, thereby missing the opportunity to apply these interventions in early childhood to improve health outcomes and cognitive functions [11,12,13,14,15,16,17,18]. 

Currently, many developmentally appropriate exergames are available for preschoolers. For example, the Wii Nickelodeon Fit—the first exergaming designed specifically for young children—represents an exciting and innovative approach aimed to help young children stay physically active while virtually engaging with their favorite cartoon characters (e.g., Dora, Diego, Kai-lan, The Backyardigans). Specifically, these exergames emphasized the improvement of motor skills, heart health, cardiorespiratory fitness, muscular strength, balance, endurance, and coordination [19]. Additionally, children were generally more physically active and had fun while playing the exergames. Yet, literature is sparse examining the effects of exergaming on preschoolers’ health-related outcomes [7] and cognition functions [11,12,13,14,15,16,17,18]. Moreover, most studies have primarily focused on physiological or psychological outcomes [10,11,12,13,14]. Such studies have not explained the effects of exergaming on other important aspects of child development in early childhood (e.g., cognitive functions, perceived competence) [20]. Additionally, some exergaming, such as Just Dance for Kids and Nickelodeon Fit, are designed specifically for young children for optimal health and cognitive development. These active video games prompt kids to jump, dance, and move as they learn core skills across reading, mathematics, science, and problem solving. Furthermore, childcare centers in metropolitan areas of China represent ideal locations to reach a vast majority of young children because parents in urban areas tend to send their children to childcare centers whiling working full-time in order to financially support their families. However, no known study has been conducted examining the effectiveness of such exergaming programs within childcare centers in China [21].

Operationally, executive functions refer to higher-order, self-regulatory cognitive processes that facilitate the monitoring and control of behavior and thinking (e.g., working memory, attentional flexibility, inhibitory control) [22]. Recent research indicates that regular PA participation and increased cardiorespiratory fitness positively impacts executive functions in children (e.g., working memory, attention, cognitive flexibility) [23,24,25,26]. For instance, gross motor skills and balance have been associated with executive functions [27] and well-developed motor skills have also been posited to be a facilitator of children’s academic capabilities in language, reading, and mathematics [28]. Research further suggests compromised motor skills in childhood to negatively and indirectly affect adolescents’ academic performances mediated by lack of PA [29]. Despite evidence linking PA, cognition, and academic achievement [23,24,25,26,27,28,29], few studies have focused on the effects of PA interventions on preschoolers’ cognitive capabilities in developing countries like China [7]. In addition, exergaming has the fun component from video games and movement component from PA, giving it multiple beneficial effects on cognition from both video games and PA. Given that video games have the potential to enhance children’s cognition per se [30], it has been proposed that exergaming may demonstrate better effects in improving executive functions compared to traditional PA. In this study, preschool children’s executive functions were assessed by cognitive flexibility skills.

As a promising child-centered PA intervention tool, the enjoyable nature of exergaming makes it an attractive PA modality in children. Additionally, exergaming also provides more autonomy and choices for children [12]. Indeed, when children play different exergaming activities simultaneously in the same room, their feelings of relatedness to their peers increases [12]. Thus, it is posited that children’s perceived competence will be greatly enhanced by exergaming play [31]. In contrast, traditional PA usually offers few choices for children because all children presumably do the same activities together at roughly the same pace under a teacher’s instructions [7]. As such, research examining whether exergaming yields better effects on executive functions and perceived competence than traditional PA is warranted. That is, it is imperative to investigate whether preschoolers’ executive function and perceived competence changes across different PA modalities (i.e., exergaming vs. traditional PA). Therefore, this study aimed to examine the effects of exergaming interventions on preschoolers’ executive functions and perceived competence as compared to a traditional PA program [7]. This study was guided by competence motivation theory that posits that successfully mastering skills will augment perceived competence, which in turn, boosts children’s actual skill performance (e.g., motor skills) and cognition [32]. Specifically, perceived competence refers to children’s self-evaluative beliefs regarding their ability to learn or accomplish certain tasks [33]. It is highly related to young children’s actual skill performance, which may result in positive cognitive development in this population [34]. 

Based on the theoretical framework and literature review, we hypothesize that: (1) Children in the exergaming group will demonstrate greater increases in executive functions as compared to their peers in the traditional PA group; and (2) children in the exergaming group may exhibit greater effects on perceived competence in comparison with those in the traditional PA program. Examining the effects of novel PA interventions like exergaming compared to traditional PA on children’s perceived competence and cognitive development will help understand how innovative PA approaches may be used in childcare centers to foster optimal development among preschool children. This project is unique in that it compares two PA interventions that promote PA and child development and investigates their effects on cognitive development and perceived competence. Findings from this research will inform the development of innovative PA programs aiming to improve health-related fitness and foster optimal development in urban preschool children in China.

## 2. Methods

### 2.1. Participants and Research Design

A total of 60 preschool children aged 4–5 years at a childcare center in a metropolitan area in Southern China were recruited to participate in this study. The childcare center was located in the downtown area of the city and served over 240 toddlers and preschool children in the 3 neighborhoods of the district. Children came from middle and upper class families in this region. Children’s socioeconomic status was determined by a brief report from the parents. The following were inclusion criteria for the sample: (1) Children aged 4–5 years; (2) children attended the urban childcare center; (3) children had no physical or mental disabilities; and (4) researchers obtained children’s parental consent. We obtained the university ethic approval and parental/guardian consent prior to the start of the study. 

We calculated the sample size with G*Power 3.1 (http://www.gpower.hhu.de/en.html), and determined that 60 children would be sufficient for 80% power to test the primary outcome variable (i.e., executive functions) while setting α = 0.05 and effect size = 0.30. This study used a quasi-experimental design with children from 4 classes (15 students/class), randomly assigned to receive the exergaming intervention or a traditional PA program with the class as the experimental unit, which was logical and reasonable in a real-world setting. The randomization allocation 1:1 with class as the block was generated by the primary researcher. During the data collection period, the childcare center offered a number of academic learning activities (e.g., math, reading, science), free games, and unstructured activities (e.g., recess) from 08:00 to 16:00 during weekdays. After baseline measurements of executive functions and perceived competence, children were assigned to either an exergaming group or traditional PA group (30 children per group). Exergaming and traditional PA were offered 20 min/session on a daily basis by trained instructors for 8 weeks. Post-intervention measures were identical to baseline measures.

### 2.2. Procedures

In general, the childcare center offered full-day programs for 4–5 year olds and young children that usually had a 20-min unstructured recess during weekdays. In other words, preschool children in this study accumulated 100 min of school recess weekly and we replaced the recess with either exergaming or traditional PA programs. Baseline and post-intervention measurements were performed in a private room within the childcare center. All children underwent identical measurements at baseline and post-intervention. In detail, participants’ executive functions and perceived competence were measured during one-on-one testing sessions. 

### 2.3. Intervention Implementation

**Exergaming Intervention Condition.** The exergaming intervention program was provided daily during the 20-min recess period 5 days/week for 8 weeks. Specifically, 4 exergaming stations were set up in an empty room within the childcare center. Each station consisted of a number of different games (e.g., Nickelodeon Fit, Just Dance for Kids, Wii sports), 1 exergame console (Wii) and related equipment (e.g., controllers), and one 32-inch TV. In detail, Nickelodeon Fit features 30 PAs integrated into interactive gameplay, targeting motor skill development, balance, coordination, cardiovascular fitness, upper and lower body strength, and core muscle strength. Some exercises included but were not limited to: Going river rafting with Diego, jumping rope with The Backyardigans, leaping over hurdles with Kai-lan, and pogo sticking with Dora. Each station could accommodate 2 to 4 children to play interactively with the exergaming system. As a result, most, if not all children were engaged and physically active during each 20-min session. 

**Traditional PA Condition.** The researchers employed a previously established traditional PA program (e.g., tag games, locomotion activities, soccer) [7] at the childcare center. The PA program encouraged preschool children to engage in structured movement activities aimed at increasing children’s engagement in PA participation. Specifically, the PA program was offered indoors or outdoors if weather permitted. For instance, children were instructed to learn several motor skills early in the week and then played games and activities with similar motor skills being embedded into the program later in the week. The games and activities targeted participants’ motor skills and varied in intensity and duration to encourage participation and enthusiasm in the sample of young children. 

**Intervention Quality.** The research team had equivalent amounts of contact with 2 PA groups through intervention interactions, weekly check-ups, and process evaluation. Weekly check-ups were conducted to ensure children received the proposed PA doses in the exergaming and traditional PA groups. As such, both groups were exposed to the same or similar amount of contact with researchers during the data collection period. Intervention quality was continuously monitored throughout the implementation of the intervention. In detail, the principal investigator met weekly with the trained instructors for program delivery to ensure consistency of the exergaming and traditional PA programs at the childcare center. Guided by the previously established protocols, the researchers conducted monitoring of weekly PA intervention implementation. The researchers also conducted process evaluation surveys to participants and program instructors on a weekly basis to monitor implementation quality and child engagement. Some sample items concerning implementation quality were, “The teachers were adhering to intervention protocol” and “No noticeable deviations are seen from protocol.” Sample items regarding child engagement included “Children generally appear to comprehend the exergaming” and “Children can play exergaming without need for excessive teacher assistance.” A 6-point Likert scale was used for both surveys. In the present study, children’s attendance at the sessions was 86% for the programs according to the records. Additionally, intervention quality was approximately 92% for the protocol components based on the weekly process evaluation survey.

### 2.4. Measures

**Demographic and Anthropometric Data.** Children’s demographic information (e.g., age, race, gender) were collected via a demographic information sheet completed by the respective classroom teachers. Children’s parents also reported their education level (e.g., high school, college, graduate, etc.), and family incomes to determine socioeconomic status. Children’s height and weight were measured at baseline in a private room using a Seca stadiometer (Seca, Chino, CA, USA) and Detecto digital weight scale (Detecto, Web City, MO, USA). 

**Figure 1:**
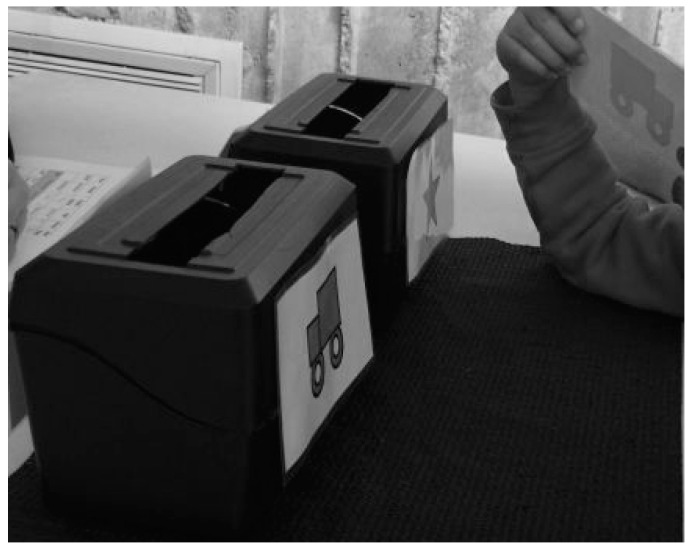
Set up for executive function test.

**Executive Functions**. In this study, participants’ executive functions were assessed by the Dimensional Change Card Sort (DCCS) Test—previously observed to be valid in the U.S. [35]. This test provides an index for the development of executive function among 3–5-year-olds, and it has been widely used in assessing individual differences in executive function for this population. In detail, preschoolers were instructed to place the laminated cards depicting various shapes and colors into black plastic boxes to assess their cognitive flexibility. They were then asked to follow the rules of increasing complexity to accurately place the cards, shift their responses in accordance with new rules, and inhibit responses which were no longer accurate once the rules changed. In detail, 2 target pictures were presented that varied along 2 dimensions (e.g., color, shape). Participants were asked to match a series of bivalent test pictures (e.g., red trucks, black stars) to the target pictures, first according to one dimension (e.g., shape) and then, after a number of trials, according to the other dimension (e.g., color). “Switch” trials were also used, in which the participant changed the dimension being matched. For instance, after 4 straight trials matching on shape, the participant was then asked to match on color and then go back to shape, hence requiring the cognitive flexibility to quickly choose the correct stimulus (see Figure 1). This test was individually administered in a private room and took approximately 5–10 min to complete, depending on the child’s cognitive levels. In general, children with higher cognitive levels would complete more tasks and thus spend more time in completing the test. Specifically, it would take 5 min if the participant could only complete steps 2–4 (color and shape games) in the standard DCCS test. However, it may take up to 10 min if the participant could complete steps 6–7 (border game) [35]. In addition, each participant was given a demonstration and practice trial for 1–2 min to make sure s/he became familiar with the testing procedures and contents. The details of the demonstration phase have been illustrated in step 2 in the instruction [35]. Scores of color and shape game performance were based upon participants who sorted 5 or more out of 6 trials correctly. Scores on the border game was based upon the number correct out of 12. More details of scoring can be found in Zelazo [35]. The internal consistency of the test was 0.75 in the present study, indicating the test had acceptable reliability among this group of children. 

**Perceived Competence.** The Pictorial Scale of Perceived Competence and Social Acceptance [36] for preschool–kindergarten assessed participants’ perceived physical competence and perceived social acceptance. Each subscale had 6 items which were scored on a 4-point scale. The item scores were averaged to provide the profiles of a child’s perception of physical competence and social acceptance. This scale was translated into Chinese and demonstrated acceptable reliability (α = 0.72) and validity in the present study. Perceived competence were individually administered in a private room in the childcare center to protect the privacy of the child.

### 2.5. Data Analysis

All data were analyzed using SPSS 22.0 (IBM Inc., Armonk, NY, USA). Descriptive analyses were conducted to describe the sample characteristics as well as the means and standard deviations of the outcomes. Second, as the preliminary analysis, 1-way analysis of variances (ANOVAs) were employed to discern if there were significant differences for the 3 outcome variables at baseline. Third, a series of 2-way (2 groups: Exergaming vs. traditional PA) ANOVAs with repeated measures (time: Baseline vs. post-intervention) were conducted to determine the differences in children’s executive functions, perceived physical competence, and perceived social acceptance. The between-subject factor was group (i.e., exergaming group vs. PA group) and the within-subject factor was time (baseline vs. post-intervention). The significance level was set at 0.05 for all statistical analyses. Effect size was assessed via Partial Eta Square (η^2^) for each comparison.

## 3. Results

The sample consisted of 30 boys and 30 girls (*M*_age_ = 4.52 years; *M*_Height_ = 106.94 ± 6.45 cm; *M*_Weight_ = 17.78 ± 2.01 kg). In detail, the sample characteristics were similar across the exergaming group (*M*_age_ = 4.65 years; *M*_Height_ = 107.78 ± 5.64 cm; *M*_Weight_ = 18.46 ± 2.10 kg), and traditional PA group (*M*_age_ = 4.38 years; *M*_Height_ = 106.10 ± 7.16 cm; *M*_Weight_ = 17.09 ± 1.91 kg) at baseline. The descriptive results of participants’ executive functions, perceived physical competence, and perceived social acceptance are presented in Table 1. There were no significant differences at baseline on executive functions, *F* = 0.14, *p* = 0.71, perceived physical competence, *F* = 0.37, *p* = 0.54, or perceived social acceptance, *F* = 0.10, *p* = 0.75. On average, participants’ executive functions increased from pre-intervention (*M* = 47.78) to post-intervention (*M* = 51.00). Moreover, children’s perceived physical competence and perceived social acceptance also increased slightly from pre-intervention (*M* = 3.09; *M* = 2.84) to post-intervention (*M* = 3.33; *M* = 3.06).

**Figure 2:**
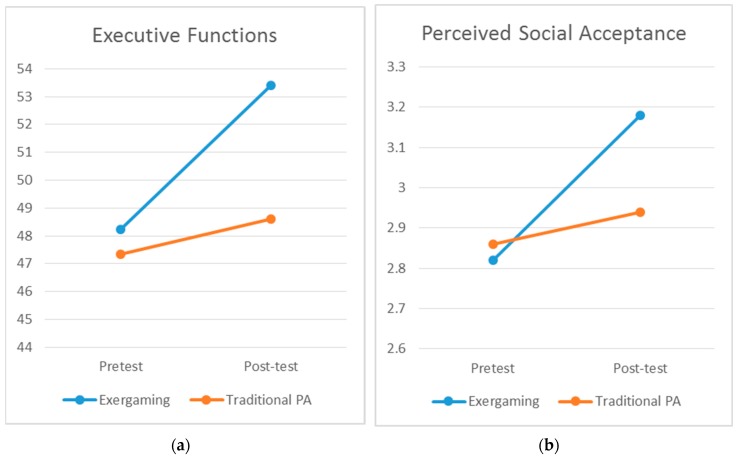
Changes of executive functions (**a**) and Changes of perceived social acceptance (**b**).

The test of interest is the time by group interaction assessed in this study. The ANOVA revealed significant time by group interaction effects for executive functions, Wilks’ Lambda = 0.83, *F*(1, 58) = 12.01, *p* = 0.01, *η*^2^ = 0.17 (see Figure 2a), and perceived social acceptance, Wilks’ Lambda = 0.91, *F*(1, 58) = 6.04, *p* = 0.02, *η*^2^ = 0.09 (see Figure 2b). Specifically, the children in the exergaming group observed significantly greater increases in executive function and perceived social support across time compared to the children in the traditional PA group. However, no significant differences were observed for the changes in perceived physical competence between the two groups, Wilks’ Lambda = 0.98, *F*(1, 58) = 1.46, *p* = 0.23, *η*^2^ = 0.03. That is, the children in the exergaming group did not display greater increases in perceived physical competence compared to the children in the traditional PA group. 

The data also indicated significant time effects for executive functions, Wilks’ Lambda = 0.64, *F*(1, 58) = 32.67, *p* = 0.01, *η*^2^ = 0.36, perceived physical competence, Wilks’ Lambda = 0.76, *F*(1, 58) = 18.04, *p* = 0.01, *η*^2^ = 0.24, and perceived social acceptance, Wilks’ Lambda = 0.79, *F*(1, 58) = 15.59, *p* = 0.01, *η*^2^ = 0.21, indicating that overall, participants’ executive functions and perceived (physical and social) competence significantly improved from baseline to post-intervention. However, group effects were not significant for executive functions, *F*(1, 58) = 2.70, *p* = 0.11, *η*^2^ = 0.04, perceived physical competence, *F*(1, 58) = 2.22, *p* = 0.14, *η*^2^ = 0.04, and perceived social acceptance, *F*(1, 58) = 0.87, *p* = 0.36, *η*^2^ = 0.02. Collectively, the data indicate that all children’s executive functions and perceived competence improved over time. 

## 4. Discussion

This study investigated the effectiveness of an innovative exergaming intervention program in improving executive functions and perceived competence in preschool children as compared to a traditional PA modality in a randomized pilot trial. The present study found that, overall, children’s executive functions, perceived physical competence, and perceived social acceptance improved over time. The findings echoed previous findings that PA has potential in enhancing children and young adults’ executive functions [37,38]. Specifically, children from both the exergaming intervention and traditional PA groups improved their executive functions over the course of eight weeks. However, mixed findings were presented in a recent review due to differences in research design, sample, intervention types, etc. [39]. It is possible that PA programs (exergaming and traditional PA) in this study led to improved executive functions. It is also plausible the improvement was due to a learning effect, such that participants’ testing scores may have improved because they learned from the baseline test. The maturation of children might be another reason for their improvement across time. Latin square research design with four groups (two more groups without baseline measurements) may be adopted to detect the confounding factors, such as learning effect in the future.

First, the results provide robust support for the first hypothesis while suggesting children in the exergaming intervention group displayed significantly greater increased scores in executive functions over time versus those from the traditional PA group. Indeed, previous empirical evidence suggests that PA could enhance executive functions (e.g., attention, working memory) in children [26,37,38]. Research evidence also indicates that PA can increase students’ concentration and mental cognition, and reduce fidgeting or other self-stimulatory behaviors [40,41]. For example, students often are more attentive and behave better after participation in PA during recess or classroom-based PA [42,43]. In a recent study with Chinese preschool children, Xiong et al. [7] found that young children in the traditional PA program had significantly increased scores on executive functions than those from the recess program (i.e., no structured PA program).

In the past decades, video games have been observed to exert positive effects on a variety of children’s cognitive outcomes, including, but not limited to, executive functions, reaction time, working memory, decision making, and spatial awareness, among others [30,44,45,46,47,48]. Furthermore, researchers recently observed exergaming to produce immediate gains in executive functions in elementary school children [49] and college students [50] as well as short-term gains in executive function in adolescents [51]. As such, exergaming—an innovative PA that combines PA and video games—may produce more benefits due to the additional positive effects of video games on children’s cognitive development as compared to the traditional PA. Taken together, it is likely that the combination of PA and video games led to the additional beneficial role of exergaming on children’s executive functioning. In addition, according to the competence motivation theory, young children’s motor skills may enhance their cognitive development. Hence, we speculate that the greater improvement in exergaming children was partially attributed to the improvement of children’s motor skills in this program over time whereas the traditional PA program in the present study simply managed to engage children in various activities. It has been posited that children’s motor skills are positively related to their cognitive development. For example, preschool children’s motor skills have been observed to be a developmental means for language acquisition [52]. Researchers have also suggested that gross motor agility and balance are positively associated with cognitive functions [53]. However, motor skills testing was beyond the scope of this study. That said, future studies may investigate preschool children’s motor skills to further investigate the mediating role of motor skills on the relationships between preschool children’s PA and cognitive development. 

In terms of children’s perceived competence, their perceived physical competence and perceived social acceptance also increased over time for both groups. In particular, the findings partially support the second hypothesis. First, the exergaming children observed significantly greater increases in perceived social acceptance from baseline to post-intervention than those in the traditional PA group. It is plausible that children in the exergaming condition worked in groups while playing exergames, which allowed more interactions with the games and peers during the intervention period (e.g., received instant feedback from exergaming after each movement), thus leading to the enhancement of their perceived social acceptance over time [18]. Exergaming children improved perceived physical competence over time, which is in line with previous findings in this area of inquiry [47,53,54,55]. However, the magnitude of enhancement of perceived physical competence failed to differ significantly between the exergaming group and the traditional PA group, indicating the exergaming program was slightly more effective in improving preschool children’s perceived physical competence than traditional PA. However, further investigation is warranted [7,49,50,56,57]. Indeed, more studies are needed to examine the approaches to effectively improve preschool children’s perceived physical competence through various PA modalities. 

The strengths of the present study lie in the adoption of innovative age and developmentally appropriate exergaming in the intervention program and objective assessment of children’s executive functions. Nevertheless, this study has several limitations that warrant readers’ attention. Firstly, the study sample was modest at best and was from only one childcare center in Southern China. The findings may not be generalized to other contexts or populations. Thus, a larger and more diverse sample of a variety of childcare centers should be targeted in the future. Secondly, the 8-week program may not be an ideal intervention duration. Therefore, future studies should adopt longer intervention lengths in preschool children in urban areas in developing countries. Additionally, there was not a true control group in this study. It is highly recommended to arrange a true control group to discern the additive effects of the PA programs over the control condition. Lastly, the present study did not assess children’s PA or energy expenditure during the data collection sessions. It is possible that young children may have various levels of PA participation during the programs. Future studies might measure these outcomes to determine the effects of various PA programs, particularly technology-based PA programs, on preschool children’s PA and health. 

The results of this study are crucial in offering implications to health professionals working with young children. Specifically, the findings suggest that structured PA programs, including exergaming and traditional PA, facilitate young children’s executive functions and perceived social acceptance. Previous studies indicated that a traditional PA program observed greater effects in improving executive function compared with a control condition [7]. Thus, structured PA programs should be encouraged as an indispensable curricular content in childcare centers. Additionally, the exergaming program has been evident in enhancing preschool children’s executive functions and perceived social acceptance in this study. That is, the results suggest that integrating an innovative, technology-based PA program into childcare curricula serves as a more effective channel in improving cognition and perceived social acceptance when compared with a traditional PA program. Although the effectiveness of exergaming on PA and other health outcomes was not determined in the present study, the findings may help inform local policy decisions and stakeholders regarding the offerings of innovative technology-based PA programs to enhance cognition and perceived social acceptance in urban childcare centers in developing countries. In summary, the findings of the present study add to the growing body of knowledge concerning PA in this population, particularly exergaming, and preschool children’s executive function and perceived competence.

## Figures and Tables

**Table 1:** Descriptive statistics of children’s executive functions and perceived competence.

	Executive Functions			Perceived Physical Competence			Perceived Social Acceptance		
	Pre-Test	Post-Test	Change Scores	Pre-Test	Post-Test	Change Scores	Pre-Test	Post-Test	Change Scores
	Mean/ SD/SE	Mean/ SD/SE	Mean/ SD	Mean/ SD/SE	Mean/ SD/SE	Mean/ SD	Mean/ SD/SE	Mean/ SD/SE	Mean/ SD
Exergaming Group (*n* = 30)	48.23/ 7.96/ 0.84	53.40/ 8.96/ 0.82	5.17 */ 4.62	3.12/ 0.41/ 0.09	3.43/ 0.46/ 0.08	0.31/ 0.51	2.82/ 0.51/ 0.09	3.18/ 0.53/ 0.09	0.36 */ 0.48
Traditional PA Group (*n* = 30)	47.33/ 10.69/ 0.75	48.60/ 10.00/ 0.78	1.27 */ 4.08	3.06/ 0.36/ 0.07	3.24/ 0.37/ 0.07	0.17/ 0.35	2.86/ 0.43/ 0.07	2.94/ 0.40/ 0.08	0.08 */ 0.38
Whole sample (*n* = 60)	47.78/ 9.36/ 0.61	51.00/ 9.72/ 0.65	3.21/ 4.75	3.09/ 0.39/ 0.06	3.33/ 0.42/ 0.07	0.24/ 0.44	2.84/ 0.47/ 0.06	3.06/ 0.48/ 0.07	0.22/ 0.45

Note: PA = physical activity; *n* = sample size; SD = standard deviation; SE = standard error, * = *p* < 0.05.
